# Engineered matrix microenvironments reveal the heterogeneity of liver sinusoidal endothelial cell phenotypic responses

**DOI:** 10.1063/5.0097602

**Published:** 2022-11-04

**Authors:** Aidan Brougham-Cook, Hannah R. C. Kimmel, Chase P. Monckton, Daniel Owen, Salman R. Khetani, Gregory H. Underhill

**Affiliations:** 1 Department of Bioengineering, University of Illinois at Urbana-Champaign, Urbana, Illinois 61801, USA; 2 Department of Biomedical Engineering, University of Illinois Chicago, Chicago, Illinois 60607, USA

## Abstract

Fibrosis is one of the hallmarks of chronic liver disease and is associated with aberrant wound healing. Changes in the composition of the liver microenvironment during fibrosis result in a complex crosstalk of extracellular cues that promote altered behaviors in the cell types that comprise the liver sinusoid, particularly liver sinusoidal endothelial cells (LSECs). Recently, it has been observed that LSECs may sustain injury before other fibrogenesis-associated cells of the sinusoid, implicating LSECs as key actors in the fibrotic cascade. A high-throughput cellular microarray platform was used to deconstruct the collective influences of defined combinations of extracellular matrix (ECM) proteins, substrate stiffness, and soluble factors on primary human LSEC phenotype *in vitro*. We observed remarkable heterogeneity in LSEC phenotype as a function of stiffness, ECM, and soluble factor context. LYVE-1 and CD-31 expressions were highest on 1 kPa substrates, and the VE-cadherin junction localization was highest on 25 kPa substrates. Also, LSECs formed distinct spatial patterns of LYVE-1 expression, with LYVE-1+ cells observed in the center of multicellular domains, and pattern size regulated by microenvironmental context. ECM composition also influenced a substantial dynamic range of expression levels for all markers, and the collagen type IV was observed to promote elevated expressions of LYVE-1, VE-cadherin, and CD-31. These studies highlight key microenvironmental regulators of LSEC phenotype and reveal unique spatial patterning of the sinusoidal marker LYVE-1. Furthermore, these data provide insight into understanding more precisely how LSECs respond to fibrotic microenvironments, which will aid drug development and identification of targets to treat liver fibrosis.

## INTRODUCTION

Chronic liver disease is a major public health concern worldwide, and its prevalence is expected to increase in coming years with a concomitant increase in incidence of liver diseases such as non-alcoholic liver disease (NAFLD) and non-alcoholic steatohepatitis (NASH).^1^ Fibrosis is one of the hallmarks of chronic liver disease and stems from an aberrant wound healing process that results in the accumulation of extracellular matrix (ECM) proteins that alter the mechanical and biochemical properties of the liver.^2,3^ While these changes in tissue properties negatively impact the behavior of many cell types in the liver, with hepatocyte dysfunction being one of the most canonical consequences, non-parenchymal cells of the liver are also prominently involved in the onset of fibrosis, and as such have been the focus of increasing interest in recent years. In particular, hepatic stellate cells (HSCs) have been implicated as significant contributors to the progression of liver fibrosis, and recent work has demonstrated that liver sinusoidal endothelial cells (LSECs) play a pivotal role in regulating HSC phenotype and coordinating signaling in the liver sinusoid microenvironment.^4,5^

LSECs are specialized endothelial cells found in liver sinusoids that play a critical role in maintaining normal liver homeostasis.^6,7^ In healthy livers, they form a semi-permeable barrier between the blood and liver parenchyma and utilize their fenestrae and abilities as scavengers to act as waste filter in the sinusoids.^8,9^ However, the onset of liver fibrosis results in the loss of characteristic LSEC fenestrae and behavior in a process known as capillarization. Also associated with capillarization is the formation of a basement membrane underneath the sinusoidal endothelium, and the dysregulation of LSEC signaling pathways, an observation that suggests LSEC capillarization, is a key mediator of fibrosis progression.^10^

In addition to their important role in maintaining balance in sinusoidal microenvironments, recent studies have provided evidence of considerable phenotypic heterogeneity in LSECs in both development and in zonation of mature sinusoids.^11–14^ Moreover, it is increasingly being demonstrated that this heterogeneity is not limited to healthy LSECs, but, in fact, exists in disease conditions such as cirrhosis and fibrosis.^15–17^ While this broad body of evidence has been accumulating, there has been much debate about the qualities and characteristics of LSECs and how best to develop a robust framework for studying LSEC phenotype.^6,8,18^ Notably, however, discussion of tissue microenvironmental factors and their influence over LSEC phenotype has largely been left out of this debate, despite their demonstrated impact on liver parenchyma and non-parenchyma.^19–21^

Indeed, given the mechanical and biochemical changes that occur in the liver, extensive work has been done to investigate the role these parameters play in influencing LSEC phenotype in both healthy and disease settings. As such, it is well established that ECM composition has a powerful influence over maintaining or altering LSEC phenotype.^22–26^ Recently, it has been shown that LSEC phenotype is also influenced by microenvironments of different elastic modulus, or stiffness, and that changes to the stiffness of the liver microenvironment can precede ECM protein deposition in fibrosis.^27,28^ Additionally, it has been shown that artificially overexpressing canonical LSEC transcription factors in non-specialized endothelial cells were insufficient in fully restoring the hallmarks of LSEC phenotype, implicating extrinsic cues from the liver microenvironment in maintaining LSEC phenotype.^29^ Despite these advances, however, current *in vitro* models of LSEC behavior and phenotype fail to fully recapitulate the full range of physiological microenvironmental stimuli needed to further interrogate the role of the microenvironment on LSEC phenotype. Specifically, it remains to be determined how ECM composition, stiffness, and secreted soluble factors cooperatively impact LSEC phenotype and heterogeneity. Additionally, current models and experimental methods utilize techniques that have limited investigatory bandwidth that restrict the scope and scale of potential inquiries. Cellular microarray platforms, however, have been shown to address these shortcomings by affording investigators the ability to thoroughly examine the influences of different microenvironmental components simultaneously on liver cells in a high throughput setting.^30–35^ As such, they are ideal for studying LSEC behavior and for performing nuanced investigations into the roles that different microenvironmental stimuli have in impacting LSEC phenotype.

In this work, we utilized a cellular microarray platform to elucidate LSEC phenotype responses and heterogeneity as a function of microenvironmental composition. Specifically, we systematically examined the combinatorial effects of variations in ECM composition, substrate stiffness, and soluble factor presence on the phenotype of human LSECs by measuring the relative expression of LYVE-1, a scavenger receptor and established LSEC differentiation marker, VE-cadherin, a highly expressed sinusoidal cadherin which is critical to LSEC function, and CD-31, a common marker of endothelia and of LSEC capillarization.^36–38^ We identified unique trends in LSEC phenotype marker co-expression as well as novel spatial patterning of these markers. We characterized the heterogeneity and plasticity of LSEC phenotype by classifying the observed responses into four unique phenotype clusters. Furthermore, we demonstrated a unique correlation between LSEC phenotype, cell contractility, and Notch signaling as a function of microenvironmental context. Taken together, these studies highlight the important role of the microenvironment on influencing canonical LSEC phenotype markers. Moreover, this work identifies the unique phenotypes that LSECs exhibit due to ECM composition, stiffness, and soluble factor alterations in models of healthy and fibrotic tissue, and contributes crucial information toward constructing a more robust framework for how LSEC behaviors should be understood and studied going forward.

## RESULTS

### LSEC phenotype markers and attachment profile respond dynamically to microenvironment context

**FIG. 1.**
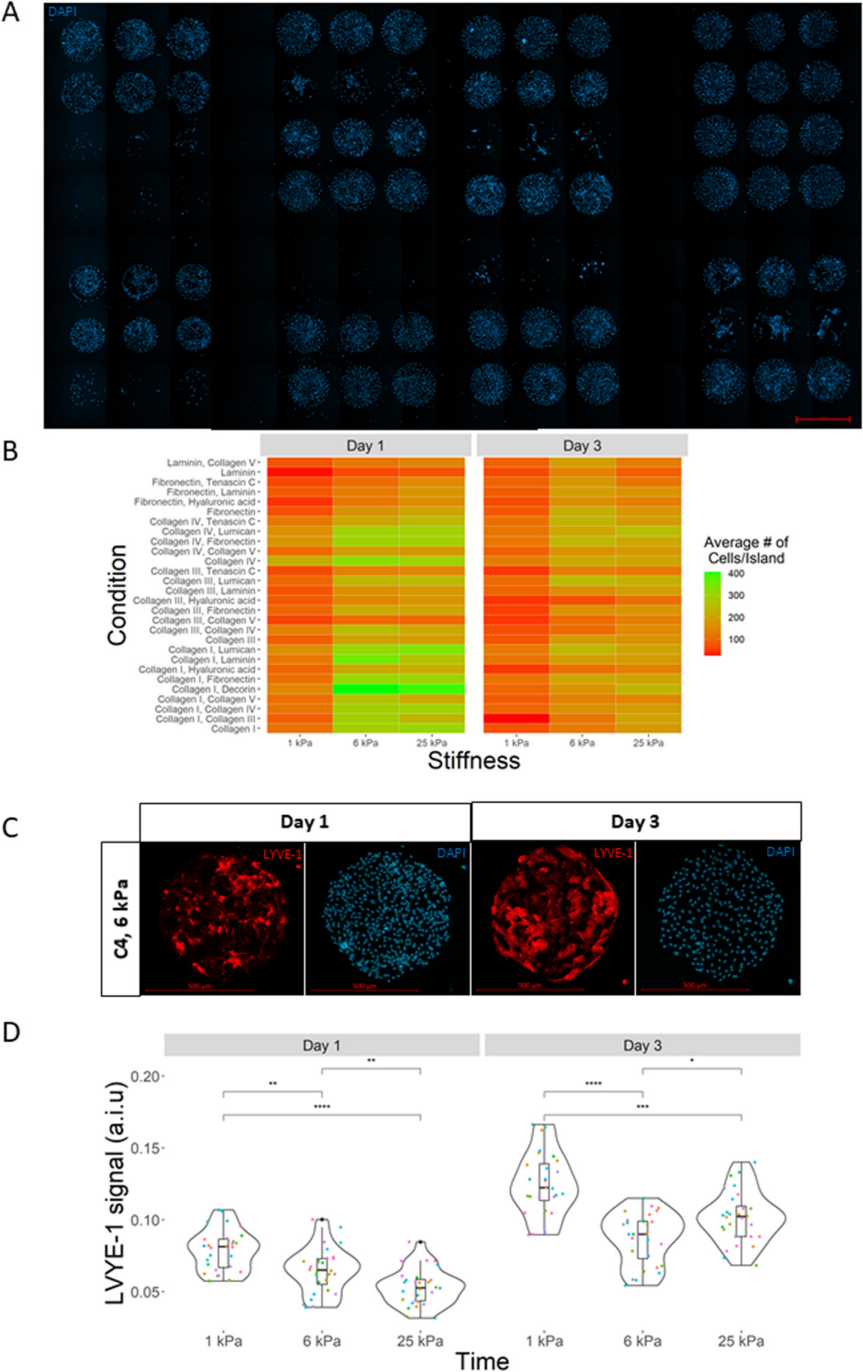
High-throughput cellular microarray analysis of LSECs. (a) Example microarray showcasing 28 unique ECM conditions on a 6 kPa substrate. (b) Heatmap of LSEC attachment after 1 and 3 days in culture, revealing the effect of ECM composition and stiffness on LSEC attachment. (c) Representative images of single condition islands showing LYVE-1 (red) and DAPI (blue). C4 islands on 6 kPa substrate at day 1 (left) and day 3 (right). (d) Box and jitter plot of LSEC LYVE-1 mean expression as a function of substrates stiffness and time. Each dot represents a unique ECM condition (28 per stiffness). Scale bars are 1000 *μ*m (a) and 500 *μ*m (c), and “ns” denotes p > 0.05, *p ≤ 0.05, **p ≤ 0.01, ***p ≤ 0.001, and ****p ≤ 0.0001.

Given the considerable evidence that LSEC behavior is strongly influenced by both ECM composition and stiffness independently,^23,27^ we sought to better understand how these two microenvironmental parameters, in various, physiologically relevant combinations, could further affect LSEC phenotype. Using a cellular microarray platform, we selected 28 ECM combinations previously implicated in influencing hepatic cell phenotype from literature,^21,23,35^ and three different hydrogel stiffnesses that mimic the mechanical properties of different stages of liver fibrosis: 1 kPa for healthy tissue, 6 kPa for early-stage fibrosis, and 25 kPa for late-stage fibrosis.^41,42^ In total, we tested 84 unique combinatorial microenvironments for their impact on LSEC phenotype. Initially, we observed that LSEC attachment profiles appear to be highly ECM dependent, with conditions containing collagen type IV (C4) exhibiting higher attachment and conditions containing laminin α1 (LN) exhibiting lower attachment. We also observed much lower cell attachment on 1 kPa substrates compared to 6 and 25 kPa [Figs. 1(a), 1(b), and supplementary material Fig. 1]. Notably, we observed that LSEC phenotypic marker LYVE-1 showed robust expression as early as 24 h into culture, and that mean LYVE-1 expression was significantly increased at the 72 h culture timepoint—particularly on 1 kPa substrates—with considerable dependence on the ECM composition [Figs. 1(c) and 1(d)].

### VE-cadherin and CD-31 expression influenced by microenvironmental composition and display junctional localization

**FIG. 2.**
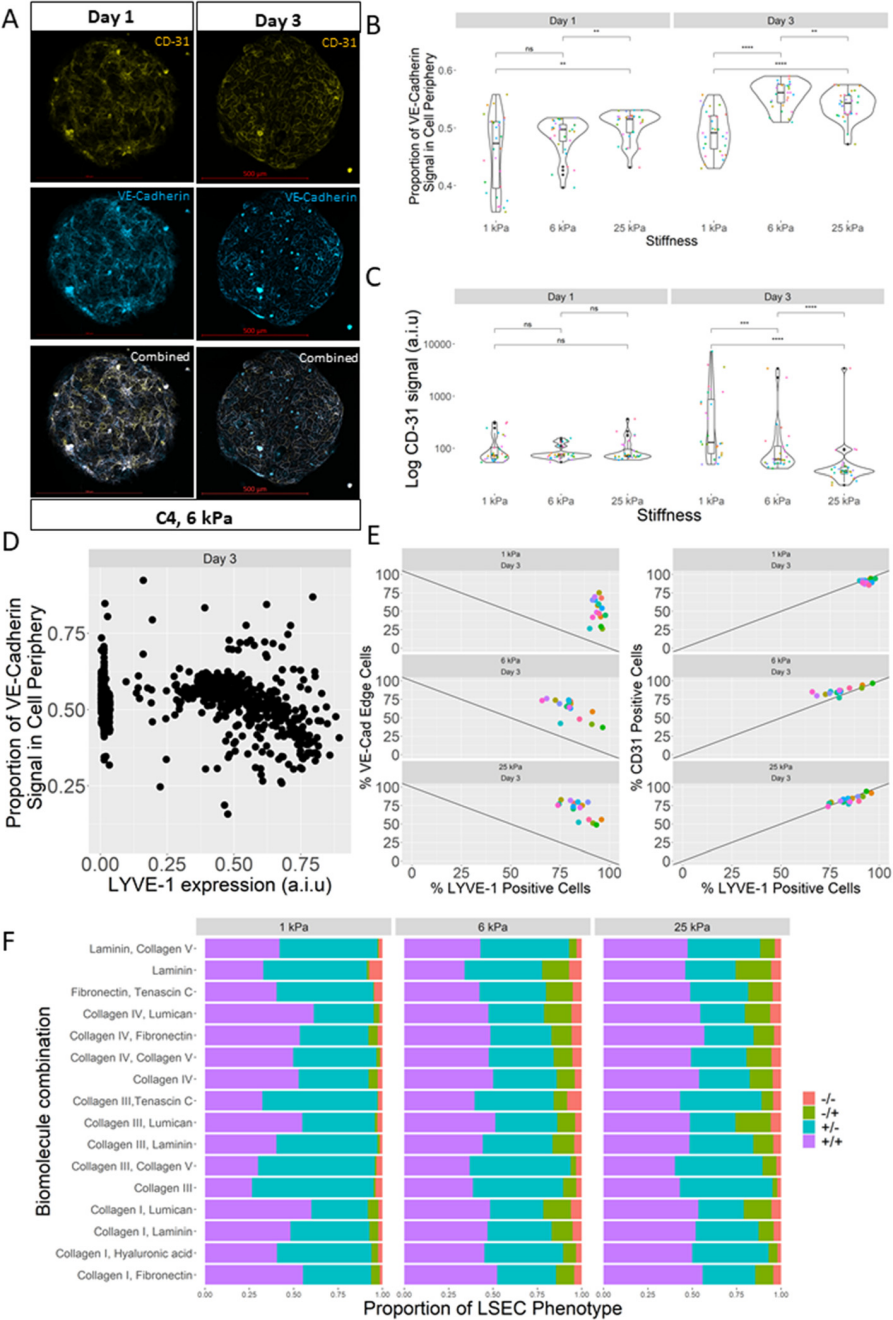
Cellular microarray data of cooperative influence of stiffness and ECM on LSEC LYVE-1, VE-Cadherin, and CD-31 expression. (a) Representative images of single condition islands showing CD-31 (yellow), VE-cadherin (light blue), and merged channel (white). C4 islands on 6 kPa substrate at day 1 (left) and day 3 (right). (b) Box and jitter plot illustrating average proportion of VE-cadherin signal detected in cell periphery per cell per condition. Each colored dot is a unique ECM combination (28 per stiffness). (c) Box and jitter plot illustrating log-scaled integrated CD-31 expression per cell per condition. Each colored dot is a unique ECM combination (28 per stiffness). (d) Scatter plot of average LYVE-1 expression vs proportion of VE-cadherin signal detected in cell periphery per cell per condition at day 3, demonstrating the sub-populations in expression. (e) Scatter plots of average number of percent LYVE-1 positive cells vs average number of percent VE-cadherin periphery localized cells at day 3 as a function of substrate stiffness (left) and of average number of percent LYVE-1 positive cells vs average number of percent CD-31 positive cells at day 3 as a function of substrate stiffness (right). Each colored dot is a unique ECM combination (16 per stiffness). (f) Stacked bar plot of relative proportions of LSEC phenotype per ECM condition per stiffness. Classification should be interpreted as LYVE-1/VE-cadherin, with – indicating negative and + indicating positive for a cell phenotypic marker (e.g., ± signifies LYVE-1 positive/VE-cadherin negative). Scale bars are 500 *μ*m, and “ns” denotes p > 0.05, *p ≤ 0.05, **p ≤ 0.01, ***p ≤ 0.001, and ****p ≤ 0.0001.

Additionally, we observed that LSEC phenotypic markers VE-cadherin and CD-31 exhibit co-expression as early as 24 h into culture. After 72 h, however, VE-cadherin and CD-31 display further changes in expression through marked junctional localization along cell periphery [Fig. 2(a)]. These signal localizations, and the degree to which they were expressed, were also observed to be dependent on microenvironmental context, with VE-cadherin and CD-31 exhibiting different response profiles. Specifically, after 72 h, LSECs demonstrated a higher junctional localization of VE-cadherin on 6 and 25 kPa vs 1 kPa substrates, while the CD-31 expression was observed to decrease with increasing stiffness [Figs. 2(b) and 2(c)].

To better understand our observations of these phenotype markers, their expression profiles were more deeply interrogated. Further inspection of the cell microarray phenotypic data revealed multimodal signal distribution profiles for each marker and consequently distinct sub-populations of cells, suggesting that a thresholding and classification system of analysis would enable improved population identification [Fig. 2(d)]. Upon introducing a cutoff threshold of expression to classify marker expression, a percent positive metric was developed for the analysis of LYVE-1 and CD-31 expressions, with a percent junction localized marker used for VE-cadherin (abbreviated as “VE-cadherin edge”). Using these metrics, phenotype marker solo and co-expression were analyzed as a function of stiffness and ECM composition. We observed that soft (1 kPa) substrates promote elevated LYVE-1 expression, and that LYVE-1 expression decreased in response to increased stiffness (supplementary material Fig. 2). Regression analysis revealed that ECM condition C1/HA positively impacted LYVE-1 expression, while C1/LU and C4/LU were determined to negatively impact LYVE-1 expression (supplementary material Fig. 3). Analysis of VE-cadherin junction localization revealed that stiff (25 kPa) substrates promoted elevated VE-cadherin junction localization, and that this effect was reduced with decreasing stiffness (supplementary material Fig. 4). Regression analysis revealed that ECM condition C1/LU positively impacted VE-cadherin junction localization, while C1/LN, C4/C5, and C1/HA were determined to negatively impact VE-cadherin expression (supplementary material Fig. 5). Interestingly, CD-31 was observed to exhibit a similar expression profile to LYVE-1 (supplementary material Figs. 6 and 7), indicating patterns of co-expression.

Intrigued by potential co-expression trends with these markers, the expression profiles of LYVE-1 positive cells vs VE-cadherin junction localized cells and LYVE-1 positive cells vs CD-31 positive cells were analyzed. Strikingly, LYVE-1 positive cells and VE-cadherin junction localized cells were observed to be inversely related, while LYVE-1 and CD-31 positive cells were observed to have a highly proportional relationship [Figs. 2(e) and 2(f)]. Moreover, on 1 kPa substrates, extreme forms of these trends were observed, with LYVE-1 positive cells establishing consistently high levels of expression independent of ECM composition. Given the unique expression trend observed between LYVE-1 and VE-cadherin, we sought to understand how ECM composition and stiffness influence populations of LYVE-1+ and VE-cadherin+ cells. Specifically, we observed that the proportions of LSECs that are positive for LYVE-1 only (+/−), VE-cadherin only (−/+), both (+/+), or neither (−/−) changed dramatically with different microenvironmental conditions. Specifically, ECM was observed to influence +/+ and +/− cell populations more on soft (1 kPa) vs stiffer (6 and 25 kPa) substrates, while influencing −/+ and +/− more on stiff (25 kPa) than softer (1 and 6 kPa) substrates [Fig. 2(e)]. Additionally, −/− and −/+ populations were observed to be highly stiffness dependent, with much higher levels of population proportionality observed on stiffer (6 and 25 kPa) vs soft (1 kPa) substrates. This microenvironmental impact is also observed when the relative expression of CD-31 is included in the heterogeneity analysis (supplementary material Fig. 8). Overall, these data highlight the influence of ECM composition and stiffness on the expression of these markers and illuminate novel LSEC phenotypic heterogeneity.

### Microenvironmental stimuli elicit spatial patterning and heterogeneity in LSEC expression of LYVE-1, VE-cadherin, and CD-31

**FIG. 3.**
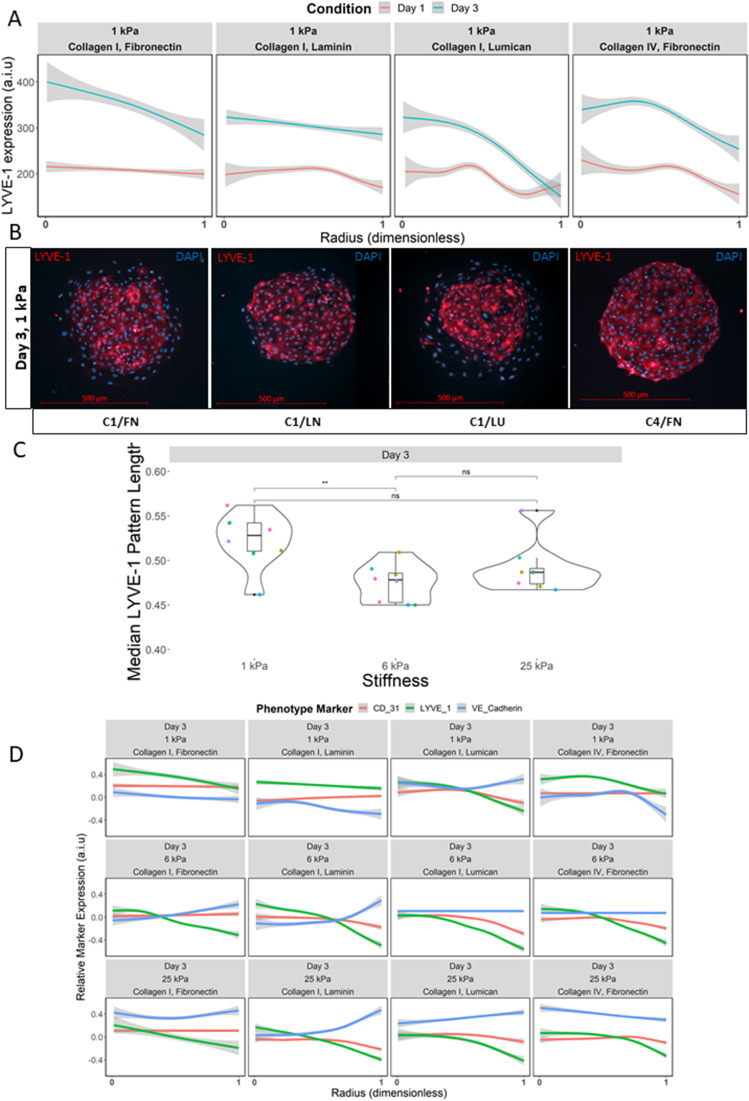
Cellular microarray data of spatial patterning of LSEC phenotypic markers as a function of ECM composition and stiffness. (a) Line plot of average integrated LYVE-1 expression on select ECM conditions on 1 kPa substrates as a function of radial distance on islands comparing day 1 expression profiles (red) to day 3 (blue). (b) Representative images of single condition islands showing LYVE-1 pattern (red) and DAPI (blue). Islands on 1 kPa at day 3 on C1/FN (far left), C1/LN (middle left), C1/LU (middle right), and C4/FN (far right). (c) Box and jitter plot illustrating median pattern length of LYVE-1 pattern as a function of ECM composition and stiffness at day 3. Each colored dot is a unique ECM combination (16 per stiffness). (d) Line plot of scaled LYVE-1 expression, scaled proportion of VE-cadherin periphery localization, and scaled CD-31 expression on select ECM conditions and substrate stiffnesses as a function of radial distance. Scale bars are 500 *μ*m, and “ns” denotes p > 0.05, *p ≤ 0.05, **p ≤ 0.01, ***p ≤ 0.001, and ****p ≤ 0.0001.

Given the unique responses these phenotypic markers exhibited as a function of their microenvironmental composition, we sought to more precisely understand these trends by down-selecting to a subset of 16 ECM conditions that showed the highest average cell attachment for follow up investigations. LSECs were then cultured on these 16 ECM microarrays, and the expression of the phenotypic markers (LYVE-1, VE-cadherin, CD-31) was quantitatively assessed following 72 h of microarray culture. Notably, LSECs at this time point displayed noticeable spatial patterning of LYVE-1 expression across the multicellular cultures that are confined to the arrayed ECM domains [Figs. 3(a) and 3(b)]. This patterning was observed to be highly stiffness dependent, with longer pattern radii that is indicative of a larger fraction of the cell monolayer expressing LYVE-1 primarily observed on 1 kPa substrates compared to 6 or 25 kPa [Fig. 3(c)]. Broadly, ECM composition was observed to establish a considerable dynamic range of pattern lengths on all stiffnesses. Linear regression modeling identified conditions containing C4 as the most impactful on altering median LYVE-1 expression radii length independent of stiffness, with C4/FN promoting longer median expression radii length, while C4/C5 promoting the opposite (supplementary material Fig. 9). Additionally, VE-cadherin and CD-31 were observed to exhibit some degree of spatial patterning as well as LYVE-1. This collective phenotypic patterning was observed to be highly dependent on both ECM composition and stiffness [Fig. 3(d)]. For example, LYVE-1 and CD-31 exhibited similar patterning for C1/LN and C1/LU, yet on C1/FN, the expression of these markers diverged, while VE-cadherin showed opposite patterning trends on C1/LU vs C4/FN.

### Relative presence of ECM proteins modulates spatial patterning and phenotypic heterogeneity in LSECs

**FIG. 4.**
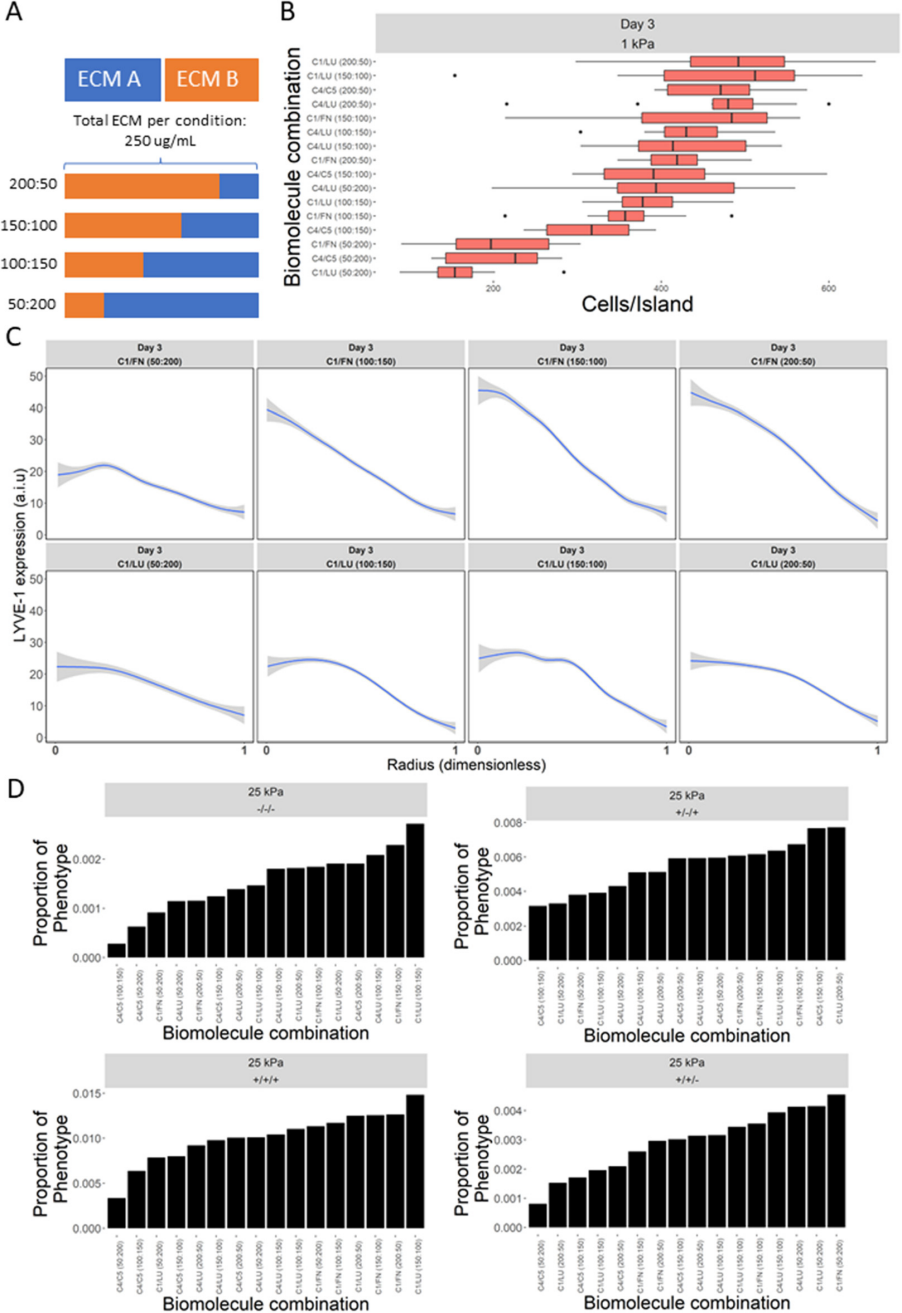
Cellular microarray data of influence of relative ECM composition on LSEC phenotype. (a) Schematic outlining combinatorial ECM conditions and the breakdown of relative ECM composition. (b) Box plot of LSEC attachment on 1 kPa substrates as a function of relative ECM composition. (c) Line plots of integrated LYVE-1 expression on select ECM conditions a function of radial distance on islands. (d) Ranked bar plots of proportions of LSEC phenotype groups as a function of relative ECM composition.

With ECM composition observed to exert such a prominent role in influencing LSEC marker expression and heterogeneity, we sought to determine the dose-responsive influence of ECM composition on LSEC phenotype. To do so, the relative concentrations of four representative ECM conditions (C1/FN, C4/LU, C1/LU, and C4/C5) were studied at the following ratios for a total of 16 conditions across the same three stiffnesses (ECM A/ECM B): 200:50, 150:100, 100:150, and 50:200 [Fig. 4(a)]. The effect of the relative composition of ECM was apparent in the resultant LSEC adhesion profiles, particularly on 1 kPa substrates [Fig. 4(b)]. Additionally, varying the ratio of components for C4/C5 conditions significantly attenuated cell attachment independent of stiffness, in contrast with C4/LU conditions which exhibited little difference in attachment regardless of relative concentration or stiffness (supplementary material Fig. 10). Most notably, the relative concentration of ECM protein was observed to substantially impact the expression and spatial patterning profile of LYVE-1. For example, while C1/LU displayed minor changes in patterning profile across different concentrations, C1/FN showed a considerable increase in pattern profile, independent of stiffness, as the concentration of C1 increased and FN decreased [Fig. 4(c)]. This was also reflected in LYVE-1 percent positive cells, with ECM condition and relative composition observed to attenuate LYVE-1 expression differently. Interestingly, while varying concentrations of C4/LU and C4/C5 elicited consistent patterning profiles independent of concentration, cells on C4/C5 generally displayed longer pattern radii, while those on C4/LU displayed shorter pattern radii (supplementary material Fig. 11). Notably, relative ECM composition was observed to have little to no impact on VE-cadherin junction localization, while the CD-31 expression was also observed to be sensitive to relative ECM composition, similar to LYVE-1 (supplementary material Fig. 12). Moreover, population heterogeneity analysis of changes in LYVE-1, VE-cadherin, and CD-31 expressions as a function of relative ECM composition revealed that ECM concentration influences the heterogeneity of expression of these three LSEC phenotype markers most dramatically on stiff (25 kPa) substrates [Fig. 4(d)]. Specifically, conditions containing LU were observed to pointedly influence LSEC heterogeneity by either positively or negatively impacting triple negative, triple positive, and double positive (−/−/−, +/+/+, +/−/+, and +/+/−) populations depending on their relative concentration and ECM combination (order of phenotype markers: LYVE-1/VE-cadherin/CD-31). Notably, +/+/− populations are least prevalent on soft (1 kPa) substrates and highest on intermediate (6 kPa) substrates, with both maintaining similar levels of −/−/− (supplementary material Fig. 12).

### Soluble factor presence influences LSEC phenotype in combination with ECM composition and stiffness

**FIG. 5.**
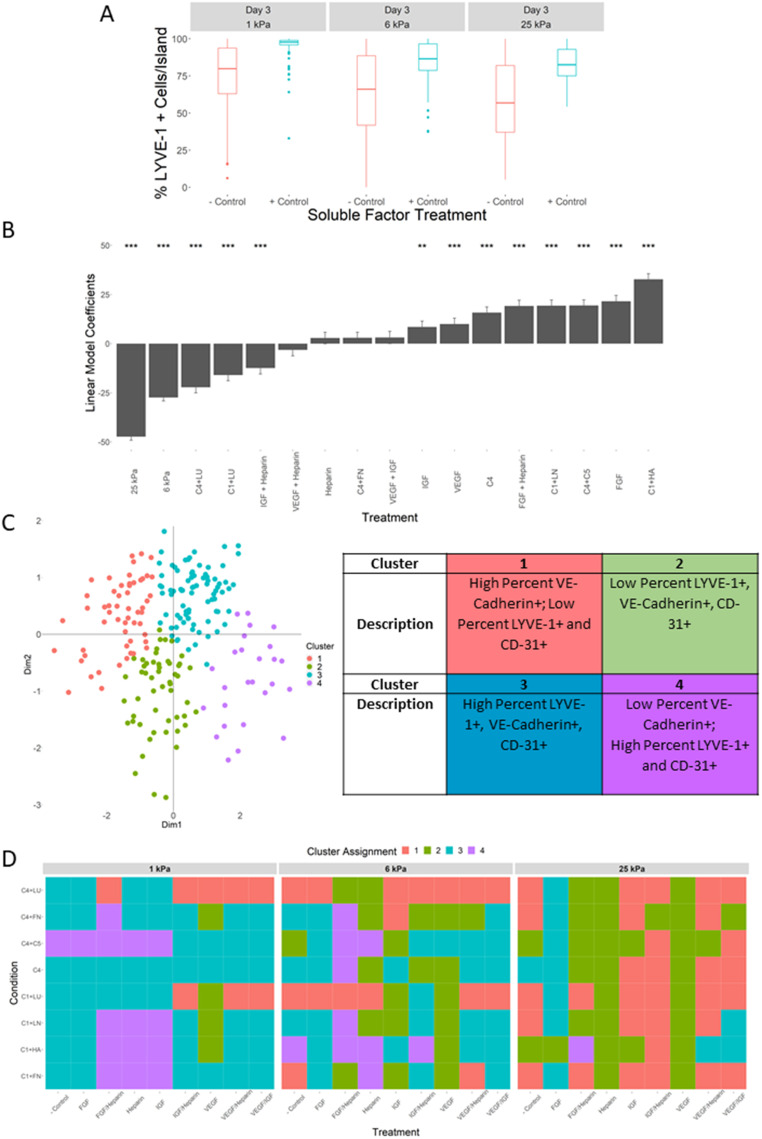
Cellular microarray data and analysis of soluble factors, substrate stiffness, and ECM composition on LSEC phenotypic heterogeneity. (a) Box plot of percent LYVE-1 positive cells with and without treatment with soluble factor cocktail at day 3 as a function of substrate stiffness. (b) Ranked bar plot of linear regression coefficients of microenvironmental components showing relative contribution to percent LYVE-1 positivity. Intercept coefficient = 133.968, Adjusted R-squared: 0.2438, F-statistic = 91.83 on 17 and 4772 DF, p-value: <2.2 × 10^−16^. (c) Scatter plot of all 216 ECM/stiffness/soluble factor conditions highlights how clusters appear relative to the principal component dimensions. Table indicates how the phenotypes sort into each cluster. (d) Heatmaps of cluster assignments of all 216 unique microenvironment conditions.

While ECM composition is an important feature of a cellular microenvironment, it is only part of the milieu of biochemical signals that interact with cells. Soluble factors such as cytokines and growth factors are also present and active in cell microenvironments, especially in the liver sinusoid. As such, we sought to understand how this facet of the cellular microenvironment impacted LSECs in combination with ECM composition and stiffness. We began by investigating the impact of soluble factors as a microenvironmental stimuli by culturing LSECs on cellular microarrays of 16 representative ECM conditions on 1, 6, and 25 kPa substrates using a base media formulation (− control, EGM, Lonza) and a cytokine supplemented media (+ control, EGM2, Lonza). Notably, we observed that the percentages of LYVE-1+ cells are significantly higher when cultured in supplemented media compared to the negative control, independent of stiffness [Fig. 5(a)]. Encouraged by this observation, we then tested eight single and two-factor combinations of four of the prominent growth factor components from the supplement media [vascular endothelial growth factor (VEGF), fibroblast growth factor (FGF), insulin-like growth factor (IGF), and Heparin] on these 16 ECM microarrays at the same three stiffness, combining for a total of 384 unique microenvironmental conditions.

We observed that soluble factor presence caused no significant reductions in LSEC attachment, and that different combinations of soluble factors could promote differential levels of attachment depending on their components, with IGF + Heparin promoting higher levels of attachment yet FGF + Heparin promoting lower levels (supplementary material Fig. 13). Regression analysis revealed that ECM composition, stiffness, and soluble factor treatment significantly impacted LYVE-1 expression, with C1/HA and FGF promoting the largest increases in percent LYVE-1 positive cells, and 25 and 6 kPa substrates promoting the largest decreases [Fig. 5(b) and supplementary material Fig. 14]. LYVE-1 spatial patterning was also observed to be impacted by soluble factor treatment (supplementary material Fig. 15). Additionally, VE-cadherin junction localization, CD-31 expression, and LYVE-1 patterning were also impacted by the combination of ECM, stiffness, and soluble factors (supplementary material Figs. 16 and 17). We also observed that while maintaining their inverse relationship, the relative proportions of LYVE-1 and VE-cadherin shifted sizably upon treatment with soluble factors, highlighting both the robust relationship between the two markers and the considerable phenotypic plasticity of LSECs (supplementary material Fig. 18). More broadly, Principal Component Analysis (PCA) combined with hierarchical clustering analysis revealed that LSEC phenotype as a function of ECM composition, stiffness, and soluble factors is remarkably heterogenous and can be characterized into four distinct populations [Figs. 5(c) and 5(d)]. Notably, all three microenvironmental stimuli were determined to be significant determinants of cluster assignment (Wilks test, p-value <0.001).

### Small-molecule inhibition of Notch and ROCK signaling pathways attenuate LSEC phenotype and spatial patterning profile

While the soluble factor experiments revealed an important role for growth factors in influencing LSEC phenotype, they typically function as agonists, affecting cell behavior by stimulating cell signaling pathways. Considering the unique trends in the data collected thus far, we sought to better understand mechanistically how and why LSECs respond to their microenvironments in such dramatic fashion. Given the demonstrated impact of these soluble factor agonists, we then considered whether pathway antagonism through small molecule inhibition could shed more light on the mechanisms regulating LSEC phenotype. Changes in tissue stiffness are hallmarks of liver fibrosis, and it is well documented that cellular contractility changes concomitantly in various hepatic cell types with increased tissue stiffness through mechanostransduction pathways.^32,34,35,43^ Additionally, Notch signaling has been implicated in LSEC capillarization *in vivo*, and activated Notch signaling has been observed in patients with cirrhosis.^44,45^ Considering the observed impact of stiffness on LSEC phenotype as well as the robust cell–cell junction formation observed from VE-cadherin and CD-31 data, we hypothesized that mechanostransduction and Notch signaling pathways were involved in regulating LSEC phenotype. To interrogate the influence of these pathways, we treated cells with gamma secretase inhibitor (GSI), which prevents the proteolytic cleavage of the Notch receptor, and thus the release of the Notch intracellular domain, and Rho-associated kinase (ROCK) inhibitor (Y-27632), which blocks myosin II activity, to better understand the role of Notch and mechanostransduction pathways in determining LSEC phenotype.

**FIG. 6.**
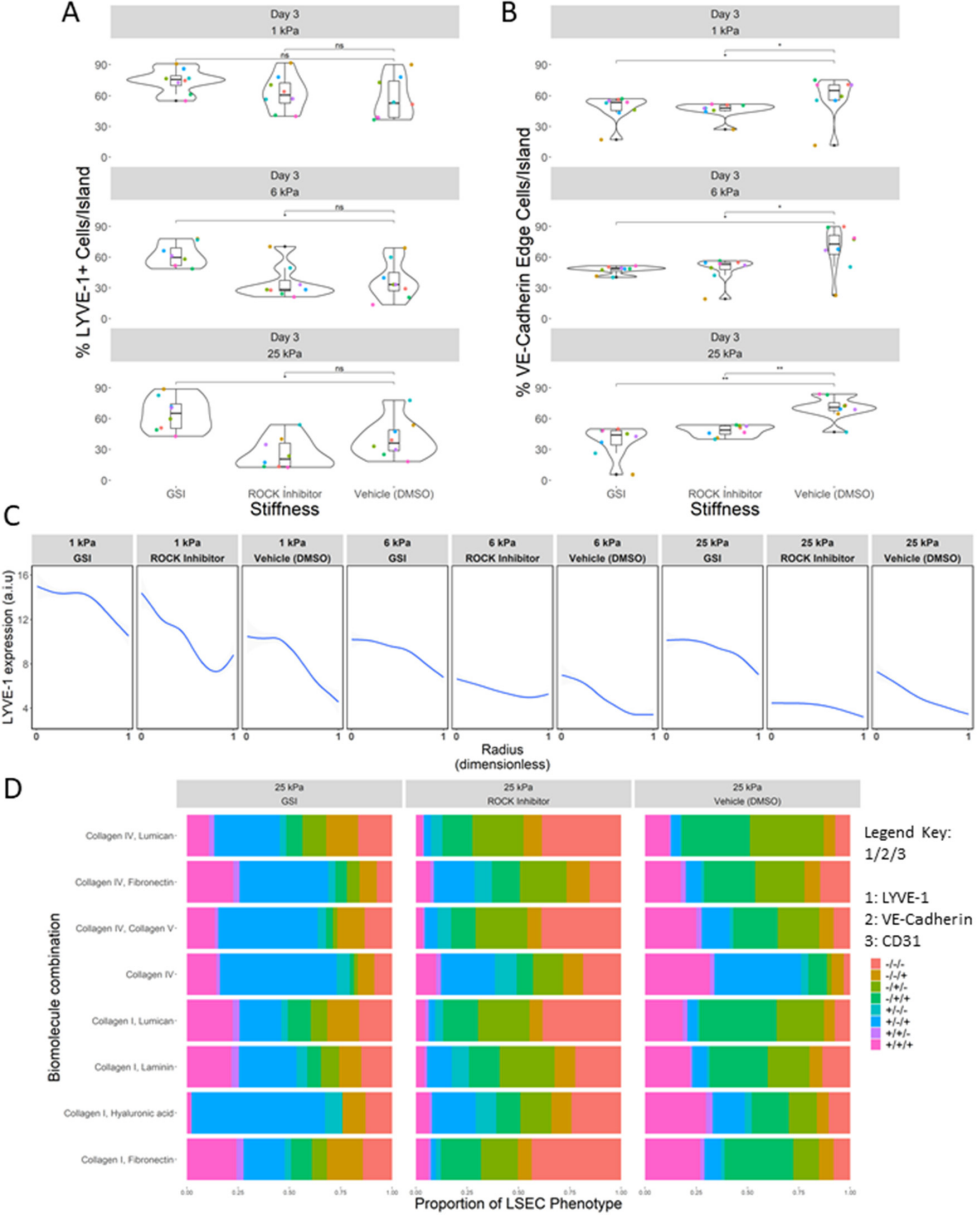
Microarray platform analysis of the impacts of contractility and Notch signaling on LSEC phenotype. (a) Box and jitter plot illustrating number of percent LYVE-1 positive cells as a function of stiffness and treatment. Each colored dot is a unique ECM combination (eight per stiffness). (b) Box and jitter plot illustrating number of percent VE-cadherin junction localized cells as a function of stiffness and treatment. Each colored dot is a unique ECM combination (eight per stiffness). (c) Line plots of integrated LYVE-1 expression as a function of radial distance on islands on 1, 6, and 25 kPa substrates with GSI, Y-27632, and vehicle control treatments. (d) Stacked bar plot of relative proportions of LSEC phenotype per ECM condition on 25 kPa substrates. Classification should be interpreted as LYVE-1/VE-cadherin/CD-31, with − indicating negative and + indicating positive for a cell phenotypic marker (e.g., +/−/+ signifies LYVE-1 positive/VE-cadherin negative/CD-31 positive).

We observed that LSEC LYVE-1 expression dramatically increased compared to control when treated with GSI, especially on stiffer (6 and 25 kPa) substrates, whereas Y-27632 treatment showed little impact [Fig. 6(a)]. Notably, LSECs treated with both GSI and Y-27632 exhibited lower VE-cadherin expression compared to control, with the largest decreases occurring on stiffer (6 and 25 kPa) substrates, while little to no effect was observed on CD-31 expression [Fig. 6(b) and supplementary material Fig. 19]. We also observed that GSI and Y-27632 treatments had a pronounced impact on LYVE-1 spatial patterning. Specifically, compared to control the treatment groups disrupted LYVE-1 spatial patterning on 6 and 25 kPa, while cells cultured on 1 kPa were less affected [Fig. 6(c)]. When the expression of all three phenotype markers was considered as a function of their ECM composition, stiffness, and small molecule inhibition, we observed that GSI and Y-27632 treatments induce a remarkable shift in phenotype proportion, particularly on stiff (25 kPa) substrates [Fig. 6(d) and supplementary material Fig. 19]. Overall, these data illuminate a previously undescribed LSEC phenotypic plasticity and underscore the role of Notch and mechanostransduction pathways in regulating LSEC phenotype.

## DISCUSSION

**FIG. 7.**
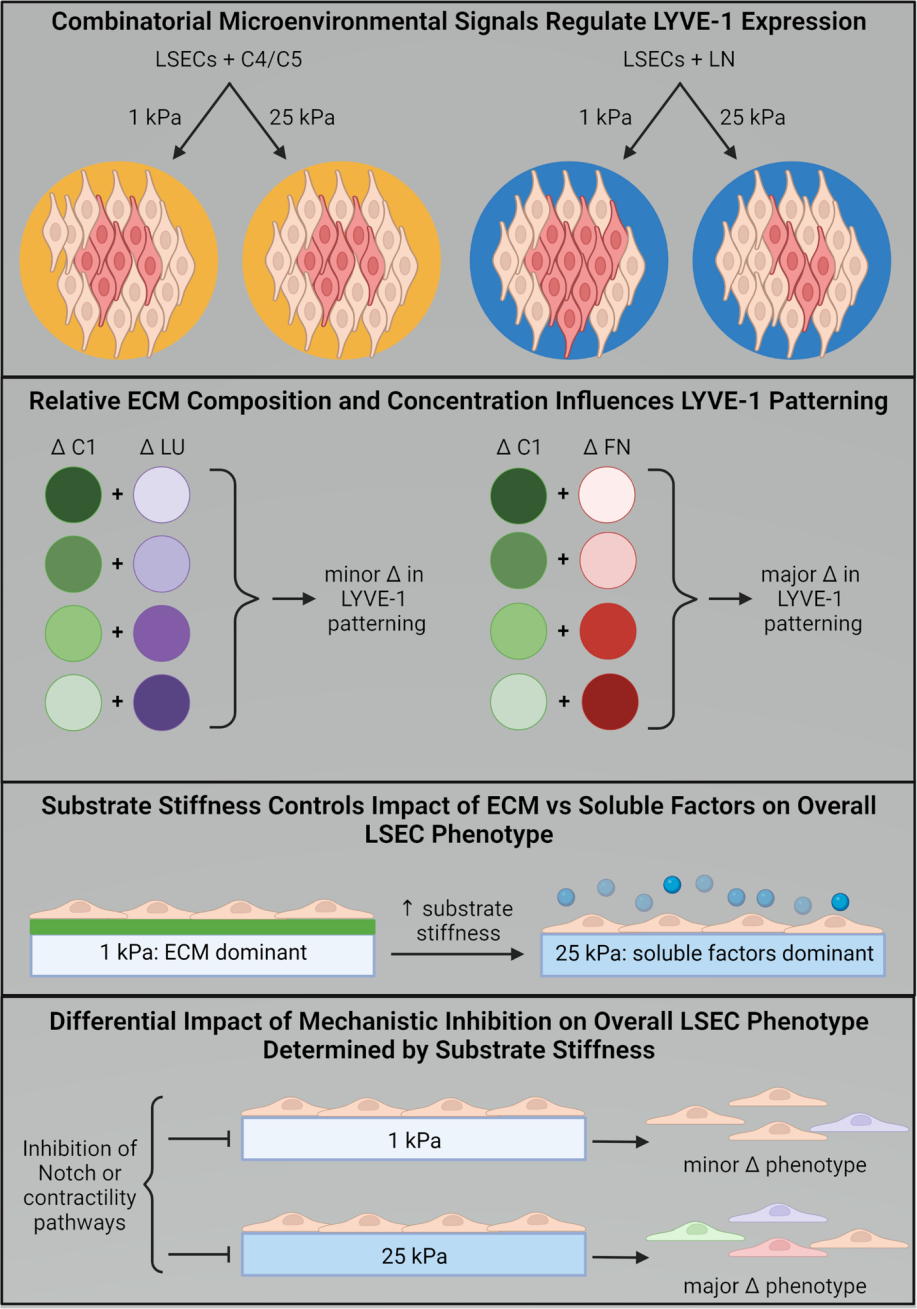
Summary figure describing the effects of cellular microenvironments on LSEC phenotype, illustrating the effect of combinatorial microenvironmental signals on LSEC LYVE-1 expression (darker red indicates increased LYVE-1 expression, top), the impact of relative ECM composition and concentration on LYVE-1 patterning (top-middle), the role that substrate stiffness plays in regulating how LSECs respond to other microenvironmental signals like ECM and soluble factors (bottom-middle), and describing the influence of substrate stiffness on mechanistic inhibition of LSECs and resulting phenotypic changes (bottom).

In this work, we demonstrate the impact that different microenvironmental stimuli have on LSEC phenotype and heterogeneity, particularly the effects these stimuli have in combination with one another (Fig. 7). For example, the influence of stiffness on LSEC LYVE-1 expression is modulated by ECM composition, with ECM conditions including laminin exhibiting strong stiffness dependence, while conditions containing proteins such as C4/C5 exhibiting less dependence on substrate stiffness. We also found that changes in the relative concentrations of ECM proteins within multicomponent mixtures regulated the spatial patterning of LYVE-1 expression within the circular multicellular domains of the cell microarray. Furthermore, substrate stiffness exhibited a cooperative influence on the responses of LSECs to exogenous stimuli, and the phenotypic responses to Notch signaling and contractility inhibitors were also modulated by the stiffness of the microenvironment. It is well documented that LSECs change their phenotype following acute liver injury in the early stages of fibrosis development through a process called capillarization. This change is characterized by a loss of fenestrae, reduced expression of canonical markers, and developing a basal membrane and has far-reaching impacts by influencing both neighboring cells of the sinusoid and immune cells more generally.^5,18,38,46,47^ Notably, similar changes in LSEC phenotype have been demonstrated in other liver pathologies, underscoring LSECs' pivotal role in maintaining liver homeostasis.^48–50^ While the local and global impact of LSEC dysregulation in the liver has been previously established, many questions about the specific roles that microenvironmental stimuli, like tissue stiffness, ECM composition, and soluble factor presence, play in this dysregulation have gone unanswered. Our data provide insight into these inquires and demonstrate the crucial role that microenvironmental signaling has on shaping LSEC phenotype.

One of the hallmarks of liver fibrosis progression is the altered mechanical properties of the tissue, namely, the increasing stiffness of the tissue microenvironment.^42^ It has been previously shown that substrate stiffness has a direct impact on endothelial cell morphology and phenotype. For example, numerous studies have reported that endothelial cells exhibit differential cell spreading, focal adhesion expression, contractility, and cell organization in a stiffness-dependent manner.^43,51–55^ Indeed, our data illustrate the complex role stiffness plays in regulating LSEC phenotype, with LSECs cultured on healthy tissue mimics (1 kPa) for 3 days consistently exhibiting elevated expression of both canonical differentiation (LYVE-1) and capillarization (CD-31) markers compared to more fibrotic mimics (6 and 25 kPa). Similar effects of stiffness with combinatorial influences of ECM composition on the expression of LYVE-1 and CD-31 and VE-cadherin junctional localization were also observed following an extended (6 day) culture period (supplementary material Fig. 20). Additionally, quantitative polymerase chain reaction (qPCR) experiments also revealed that LSECs expressed higher mRNA expression levels of KLF2, eNos, and SE-1 on soft (2 kPa) vs stiff (25 kPa) substrates, while STAB1 and kit expression levels were found to be comparable (supplementary material Fig. 21). Moreover, stiffness has a unique influence over LSEC heterogeneity, as exemplified in the relative occurrence of both the triple-positive (+ for LYVE-1, VE-cadherin, and CD-31) and triple-negative (− for LYVE-1, VE-cadherin, and CD31) phenotypes. Specifically, there is little difference in the proportions of triple-positive category between stiffnesses, yet there is a dramatic difference in the proportions of the triple-negative category between stiffnesses, with a larger share observed on stiffer (6 and 25 kPa) substrates, underscoring the major role stiffness plays on shaping the population of this phenotypic category. More broadly, when PCA and hierarchical clustering were performed to parse out the specific effects of the different stimuli, we observed that cluster assignment appears strongly influenced by ECM composition on healthy tissue mimics (1 kPa), whereas soluble factor presence appears as a more dominant influence cluster assignment on fibrotic mimics (25 kPa).

In addition to changes in mechanical properties, fibrogenesis also alters the ECM composition of the liver sinusoid. It is well established that concomitant with LSEC capillarization is the formation of a basement membrane, comprised of ECM proteins like C4, FN, and LN, and that as fibrosis progresses, the ECM composition of the liver shifts with increased levels of C1 and C3.^56–59^ Recent studies have indicated that LYVE-1 expression is reduced *in vivo* in late-stage fibrosis and cirrhosis, a trend which we do broadly see in our data.^36,60^ However, important LSEC–ECM interactions have been well documented, with ECM composition shown to impact LSEC phenotype via altered rates of survival, ECM receptor expression, and other phenotypic characteristics.^23,26,61–65^ Our data provide previously undescribed levels of detail regarding the influences ECM composition has on LSEC phenotype, particularly with LYVE-1 expression overall and the unique spatial patterning of LYVE-1. LYVE-1 expression radii were found to be highly ECM dependent on soft (1 kPa) substrates vs stiffer (6 and 25 kPa) substrates, and that ECM conditions containing C4 generally promoted longer LYVE-1 expression radii. Strikingly, when we interrogated the effect of relative ECM compositions on LYVE-1 patterning, we observed some formulations promoted changes in both the percentage of LYVE-1 positive cells and LYVE-1 radial pattern length regardless of stiffness, such as C4/C5, while with others such as C1/FN, we influenced LYVE-1 expression but in a much more stiffness dependent manner.

Interestingly, changes in the relative composition in C1/LU and C4/LU did not appreciably impact the percentage of LYVE-1 positive cells or LYVE-1 radial pattern length. When considering ECM effects on LSEC phenotype more generally, linear regression analysis revealed that C4/LU simultaneously has a negative impact on percent LYVE-1 positive and CD-31 positive while promoting a longer LYVE-1 median expression radii length. However, while C1/HA was the strongest promoter of percent LYVE-1+ and CD-31+, it had no significant impact on LYVE-1 expression spatial patterning. Additionally, PCA and hierarchical clustering analysis revealed that C4 was consistently classified into cluster three, which is classified as exhibiting elevated levels of all three phenotypic markers. C4 is a prominent component of the basement membrane and, thus, can be associated with capillarization, and C1 is a classic component of fibrotic tissue and, therefore, of fibrosis. LU is known to be upregulated in NASH and has been shown to be critical in fibrosis progression, and HA is a known marker of liver fibrosis and is processed by LSECs as part of their scavenger functionality.^66–69^ Additionally, it has been shown that endothelial cells exhibit higher levels of traction on the periphery of ECM islands compared to the center, as measured by traction force microscopy (TFM), while others have demonstrated that altering ECM composition and stiffness can impact traction force magnitudes.^35,43,70^ Additionally, it has been reported that endothelial cell permeability is modulated by substrate stiffness through changes in cell-ECM traction stresses and cell–cell junctional tensions, with cells on soft substrates (1 kPa), promoting higher cell-cell junction integrity compared to stiffer substrates (11 kPa).^51^ Our data support these findings and indicate that LSEC contractility can be modulated by microenvironmental context, and that relative LSEC contractility regulates LSEC phenotype. These observed influences of ECM composition and substrate stiffness on LYVE-1 patterning suggest that the canonical loss of LYVE-1 expression during fibrosis may not be equally experienced by LSECS in the liver sinusoid, and that relative LYVE-1 expression may require specific interplays between biochemical and mechanical forces. Furthermore, regarding the role of LU and HA in LYVE-1 expression, our data demonstrating how the relative presence of LU considerably impacts LSEC phenotype strongly suggest that LU plays a more prominent role in the regulation of cell phenotypes during the progression of fibrosis in the liver sinusoid than previously known, while the impact of HA on LYVE-1 expression is especially intriguing given HA's role as an important component of fibrotic tissue in the liver. Taken together, these findings illustrate the complexities of LYVE-1 expression in different stages of fibrosis and suggest that directly associating the relative stage of liver fibrosis with a particular level of LYVE-1 expression may not always be appropriate.

Another key microenvironmental component in the liver sinusoid is the presence and activity of soluble factors in the form of cytokines and growth factors. It has been previously demonstrated that LSECs are involved in both secreting and responding to soluble factors in both autocrine and paracrine pathways, and these interactions with soluble factors are key to regulating LSEC phenotype.^38,46,71–75^ Our data corroborate this critical role that soluble factors play in shaping LSEC phenotype and further show that soluble factors in combination with stiffness and ECM composition can produce a wide range of phenotypes. Interestingly, treatment with heparin, a thrombin antagonist, was observed to have mild positive effects on LYVE-1 and CD-31 percent positivity, but only on softer (1 and 6 kPa) substrates, yet strong negative effects on VE-cadherin junction localization on all stiffnesses. Furthermore, there is growing evidence that there is crosstalk between VE-cadherin and VEGF receptors, and this signaling is critical for endothelial mechanostransduction.^76,77^ Our data underscore this dual sensitivity of VE-cadherin to both stiffness and treatment with VEGF, corroborating previous findings regarding their conjugal mechanistic relationship.

Mechanostransduction signaling pathways have been implicated as key regulators of endothelial cell phenotype and function.^78^ In this study, treatment of LSECs with the ROCK inhibitor, Y-27632, had a varied impact on LSEC phenotype. Specifically, LYVE-1 and CD-31 expressions were largely unaffected by Y-27632 treatment on softer (1 and 6 kPa) substrates, compared to control, but their expression levels were observed to decrease on stiff (25 kPa) substrates. Interestingly, VE-cadherin junction localization decreased, compared to control, in response to Y-27632 treatment, independent of stiffness. Moreover, ROCK inhibition had no impact on LYVE-1 spatial patterning on soft (1 kPa) substrates but was observed to abrogate the expression of LYVE-1 across the multicellular cultures as substrate stiffness increased. Taken together, these substrate-dependent responses to Y-27632 treatment suggest that LYVE-1 spatial patterning within confluent multicellular domains is, indeed, linked to ROCK signaling, and to LSEC contractility more broadly, and that LSEC mechanostransduction signaling pathways are strong candidates for potential therapeutics and treatments.

One of the key findings from this work was the observed relationships among LYVE-1, VE-cadherin, and CD-31. Specifically, scatter plots of LYVE-1 and CD-31 percent positivity as a function of ECM composition, stiffness, and soluble factor treatment reveal a positively proportional relationship between the two markers, while similar plots of LYVE-1 percent positivity and VE-cadherin junctional localization reveal an inversely proportional relationship. CD-31 has been implicated in regulating mechanostransduction pathways in endothelial cells, and experiments testing the mechanosensing capabilities of LSECs have shown that VE-cadherin and CD-31 colocalize along with vascular endothelial growth factor receptor 2 (VEGFR2) to form a mechanosensory complex. Intriguingly, it has been shown that the introduction of shear stress across endothelial cells triggered a decrease in tension on VE-cadherin, while simultaneously increasing tension across CD31, and that static cultures of endothelial cells on substrates of increasing stiffness enhanced VE-cadherin-mediated forces.^51,79^ Finally, previous work investigating the vascular endothelium of human and rat liver sinusoids has revealed striking LYVE-1/VE-cadherin colocalization.^37,76,80^ LSEC capillarization is at the center of many debates about LSEC phenotype, with relative LYVE-1 and CD-31 expressions often cited as evidence of LSEC phenotype and identity, yet capillarization has been described as a gradual process, with some LSECs observed undergoing “pseudocapillarization” prior to capillarization.^36,46,81^ The cell microarray findings reported here highlight the complexity of such phenotypic transitions in response to microenvironmental conditions. For example, mimicking the early transition from healthy LSEC to pseudocapillarized, as modeled by culturing LSECs on 1 vs 6 kPa substrates, respectively, may represent an inflection point during which the single LSEC marker expression trends, as well as co-expression trends, of LYVE-1, VE-cadherin, CD-31 expression, and patterning begin to shift and become more influenced by ECM composition and soluble factors. Previous work has identified 6 kPa as promoting a pseudocapillarized LSEC phenotype, and our data corroborate this observation.^27^

## CONCLUSIONS

Overall, these findings obtained using a cellular microarray approach to mimic with high fidelity and throughput the different stages of liver fibrosis reveal a range of unique LSEC–microenvironment interactions. This new understanding of the heterogeneity of LSEC phenotype, and how these phenotypes are direct functions of microenvironmental inputs, can serve as an important guide to understanding of how LSEC differentiation or capillarization is maintained or altered in the dynamic liver sinusoidal microenvironment during fibrosis. While this work reveals insights into the microenvironmental impacts on LSEC phenotype, investigating additional phenotypic markers and assaying other LSEC behaviors as functions of microenvironmental context are still required to help achieve a more complete understanding of how LSECs influence and are influenced by liver fibrosis and should be the focus of future studies. Furthermore, future *in vitro* studies into NAFLD and NASH at the tissue level would also benefit from the transition from single cell type-centric models to multiple cell type models, particularly through the incorporation of non-parenchymal cell types like hepatic stellate cells, which are inextricably linked to LSECs and fibrosis progression. Understanding how LSECs respond to other sinusoidal cell types, in potential cooperation with the ECM effects examined here, will be crucial toward the further identification of the microenvironmental triggers and dampeners of pro-fibrotic feedback loops in the liver sinusoid.

## METHODS

### Cell culture

For all experiments in this study, human liver sinusoidal endothelial cells were used at passage 15 [Sciencell Research Laboratories (Carlsbad, CA)]. For passaging, cell culture flasks of tissue culture plastic were coated with fibronectin (0.03 mg/ml) for no less than 4 h prior to seeding, and cells were then seeded subsequently cultured under controlled environmental conditions (37 °C and 5% CO_2_). Cells were treated with trypsin-Ethylenediaminetetraacetic acid (EDTA) (0.25% v/v) for 5 min to detach them for sub-culturing. For passaging cells and for culturing cells on arrays (unless otherwise specified), EGM Endothelial Cell Growth Medium Bulletkit (Lonza, CC-3124) was used, which contains EBM^TM^ Basal Medium (Lonza, CC-3121) and EGMTM Endothelial Cell Growth Medium SingleQuots^TM^ Supplements (Lonza, CC-4133). For soluble factor experiments, EMG2 Endothelial Cell Growth Medium-2 BulletKit (Lonza, CC-3162) was used and is comprised of EBM^TM^-2 Basal Medium (Lonza, CC-3156) and EGM-2 SingleQuots Supplements (Lonza, CC-4176). For individual and combination soluble factor treatments, VEGF was delivered at 0.5 ng/ml, FGF (bFGF) at 10 ng/ml, IGF (R3-IGF-1) at 20 ng/ml, and heparin at 22.5 *μ*g/ml.

For microarray experiments, cells were seeded on arrays at 2E5 cells per slide. For immunocytochemistry experiments, cells were left to adhere on arrays for 30 min, after which arrays were washed twice with 1× phosphate buffered saline phosphate buffered saline (PBS), and then experiment-specific treatments were delivered. All drugs used in these experiments were prepared and reconstituted according to the manufacturer's instructions.

### Preparation of polyacrylamide hydrogels

Polyacrylamide (PA) hydrogels were prepared using previously described methods and protocols.^32,39^ Briefly, glass microscope slides (25 × 75 mm^2^) were washed with 0.25% v/v Triton X-100 in dH_2_O for 30 min on an orbital shaker. The slides were then etched with 0.2 N NaOH for 1 h, rinsed with dH_2_O, sprayed with compressed air, and placed on a hot plate at 110 °C until completely dry. Slides were then silanized by submerging them in 2% v/v 3-(trimethoxysilyl) propyl methacrylate in ethanol and placed on the shaker to react for 30 min. Silanized slides were then washed with ethanol on the shaker for 5 min, sprayed with compressed air, and dried on the hot plate at 110  °C. PA hydrogels with defined elastic moduli were fabricated using three prepolymer solutions with different acrylamide/bis-acrylamide percentage weight/volume ratios to achieve final elastic moduli of 1 kPa (4% acrylamide and 0.4% bis-acrylamide), 6 kPa (6% acrylamide and 0.45% bis-acrylamide), and 25 kPa (8% acrylamide and 0.55% bis-acrylamide), each with similar porosity.^40^ A 20% w/v solution of Irgacure 2959 (BASF, Corp.) in methanol was mixed with each of these prepolymer solutions to achieve a final working solution with a prepolymer:Irgacure ratio of 9:1 (Irgacure diluted at 1:10 in final working solution). 100 *μ*l of this working solution was then placed onto silanized slides and sandwiched with cover glass (22 × 60 mm^2^). The slides containing working solution and cover glass were then transferred to a UV oven and exposed for 10 min to 365 nm UV A light (240E3 *μ*J). After polymerization, the cover glass was removed from the gel, and slides were submerged in dH_2_O at room temperature for 72 h to remove excess reagents from the hydrogels. Before microarray fabrication, hydrogel substrates were dehydrated on a hot plate for at least 15 min at 50 °C.

### Array fabrication

Cellular microarrays were fabricated using previous protocols.^30–35^ Briefly, ECM proteins for arraying were diluted in a 2× growth factor buffer solution comprised of 38% v/v glycerol in 1× phosphate-buffered saline (PBS), 10.55 mg/ml sodium acetate, 3.72 mg/ml EDTA, and 10 mg/ml 3-[(3-cholamidopropyl)dimethylammonio]-1-propanesulfonate (CHAPS), and subsequently deposited in a 384-well V-bottom microplate. Unless otherwise specified, all single ECM solutions were prepared at a final concentration of 250 *μ*g/ml, and two-factor ECM solutions were prepared at 125 μg/ml per ECM, with dH_2_O as the diluent to achieve a final volume of 10 *μ*l per microwell. To transfer ECM condition solutions from the source plate to PA hydrogel substrate, a robotic benchtop microarrayer (OmniGrid Micro, Digilab) loaded with SMPC Stealth microarray pins (ArrayIt) was used, which produced array islands of ∼600 *μ*m diameter. After fabrication, arrays were stored at room temperature and 65% relative humidity overnight and then left to dry at room temperature and standard humidity in the dark. Prior to adding cells, all arrays were sterilized with 30 min UVC while submerged in a 1× PBS solution containing 1% v/v P/S.

### Image processing and microarray analysis

Cellular microarrays were imaged at 10× magnification. Images of entire arrays were converted to eight-bit TIFF files per output channel by Fiji (ImageJ version 1.51n). To identify nuclei for cell counts and regions marked by fluorescence, the IdentifyPrimaryObjects and IdentifySecondaryObjects modules of CellProfiler (version 2.2.0) were used, and single-cell fluorescence intensity was quantified using the MeasureObjectIntensity module. Fractional junction localization was quantified using the MeasureObjectIntensityDistribution module. Dextran-rhodamine marker islands were used to identify the location of arrayed conditions within each image.

### Immunocytochemistry

Samples were fixed in a 4% w/v paraformaldehyde prepared in 1× PBS for 15 min. Fixed samples were then permeabilized with a 0.25% v/v Triton X-100 solution in 1× PBS for 10 min and incubated in a 5% v/v donkey serum and 0.1% v/v Triton X-100 blocking buffer in 1× PBS for 1 h at room temperature (25 °C). Primary and secondary antibodies were prepared in blocking buffer comprised of 0.1% w/v bovine serum albumin (BSA) and 0.1% v/v Triton X-100 blocking buffer in 1× PBS, and after blocking, samples were incubated overnight at 4 °C with one or both primary antibodies in blocking buffer. Samples were subsequently washed with 1× PBS and then incubated for 1 h at room temperature with one or both secondary antibodies diluted in blocking buffer (supplementary material Table 1). Finally, samples were mounted in Fluoromount G with 4′,6-diamidino-2-phenylindole (DAPI) (Southern Biotech, 0100–20) and imaged using an AxioScan Z.1 (Carl Zeiss, Inc), with associated Zen Pro software and Cytation 5 (Agilent Technologies) no sooner than one day after mounting.

### Statistical testing

All array experiments consisted of at least three biological replicates, with four technical replicates, or islands, per biological replicate for 28 ECM arrays and 12 technical replicates per biological replicate for eight ECM arrays per combination of arrayed condition, treatment, and readout. Linear regression analyses were performed using the base lm() function in R and homoscedasticity, normal distribution of residuals, and the absence of leveraged outliers was confirmed for each model using residual-fit, Q–Q, and scale-location plots (R Core Team, 2017, R Foundation for Statistical Computing). The outputs of each regression are presented as coefficient estimates and associated standard error. Coefficients in regressions represent mean changes in percentage of cells positive for the marker in question for LYVE-1 and CD31 experiments and represent mean changes in percentage of cells with junction localized signal for VE-cadherin positive experiments. For LYVE-1 pattern regression, coefficients represent mean changes in median pattern length per island, and for cell attachment regression, they represent mean changes in number of cells per island. For comparison between conditions in this study, non-parametric Wilcoxon tests were performed using the stat_compare_means function from the “ggpubr” package in R. P values of <0.05 were considered significant for all statistical comparisons.

## SUPPLEMENTARY MATERIAL

See the supplementary material for plots of full datasets and analysis regarding the expression of LSEC phenotype markers LYVE-1, VE-cadherin, and CD-31 as a function of ECM composition, ECM concentration, stiffness, soluble factor treatment, and drug treatment, as well as plots of LSEC mRNA expression, table of antibodies used, and table of ECM notation.

## Data Availability

The data that support the findings of this study are available from the corresponding author upon request.
